# Decreased absolute numbers of CD3^+^ T cells and CD8^+^ T cells during aging in herpes zoster patients

**DOI:** 10.1038/s41598-017-15390-w

**Published:** 2017-11-08

**Authors:** Li Wei, Jianguang Zhao, Wei Wu, Yu Zhang, Xuyan Fu, Lifeng Chen, Xiaoting Wang

**Affiliations:** 1 Collaborative Innovation Center for Diagnosis and Treatment of Infectious Diseases, State Key Laboratory of Diagnostic and Treatment of Infectious Diseases, The First Affiliated Hospital, Zhejiang University School of Medicine, Hangzhou, China; 2 Medical Oncology, The First Affiliated Hospital, Zhejiang University School of Medicine, Hangzhou, China; 3 Department of Dermatology, The Dermatovenereology Hospital, Quzhou, China; 4 Department of Dermatology, The First Affiliated Hospital, Zhejiang University School of Medicine, Hangzhou, China; 5 Department of Dermatology, The First Affiliated Hospital, Zhejiang University School of Medicine, Hangzhou, China

## Abstract

Herpes zoster (HZ) is an infectious dermatosis with high incidence worldwide. Age is a key risk factor for HZ, and postherpetic neuralgia (PHN) is the main sequelae. Until now, no index has been available to predict the pathogenesis of PHN, and rare reports have focused on the immune response during aging and PHN. In this study, we selected immunoglobulin and complement proteins as markers for humoral immunity, while T lymphocyte subsets and natural killer (NK) cells were selected as markers for cell immunity, to systematically study the characteristics of immune responses in the peripheral blood of HZ patients. Our data showed that the absolute number of CD3^+^ T cells and CD8^+^ T cells decreased during aging and PHN. This implies that more attention should be paid to prevent the occurrence of PHN, especially in the aged population.

## Introduction

Herpes zoster (HZ), a distinctive syndrome induced by reactivation of varicella zoster virus (VZV), is primarily an infectious dermatosis with a high incidence of sequelae, and it requires long-term treatment^1–3^. Increasing evidence indicates that HZ has a serious effect on patient quality of life and increases medical burden.

HZ occurs frequently in individuals with immune deficiency and/or suppression^4–6^. During childhood, VZV remains latent in the ganglia of sensory nerves throughout the early years of the host. Age is the key risk factor for the pathogenesis of HZ^7^. Additionally, age has been reported to be closely related with the onset of postherpetic neuralgia (PHN), a severe sequelae of HZ. However, the mechanism of how age is involved in the PHN remains unknown^8–11^. Unfortunately, only a few studies have focused on immune responses during aging and PHN.

In this study, we aimed to investigate the characteristics of immune responses during PHN in HZ patients of various ages by detecting the T lymphocyte subsets, immunoglobulin and complement proteins, as well as plasma C-reactive protein (CRP) levels.

## Materials and Methods

### Patients

One hundred ninety-two HZ patients (female: 94, male: 98; age: 57.69 ± 14.42 years) who were hospitalized in our department between January 2010 and September 2015 were recruited for this study. Another 27 HZ patients (female: 12, male: 15; PHN^+^: 9, PHN^−^: 18; age: 67.74 ± 7.43 years) who were hospitalized in our department between July 2017 and August 2017 were recruited to study the different phenotypes of CD8^+^ T cells in this study. Fourteen age- and gender-matched individuals who received physical examinations served as controls. HZ was diagnosed by experienced dermatologists based on clinical features using the ICD-10 code B02 (Zoster). The exclusion criteria were as follows: patients with systemic diseases (e.g., cardiac and hepatic disorders), malignant tumors, and autoimmune diseases (e.g., systemic lupus erythematosus); patients who received an immunosuppressant; and women who were currently pregnant and breastfeeding.

### Study design

Patients were divided into three groups by age, including young age (aged ≤40 years), the middle age (aged >40 years and <60 years), and old age (aged ≥60 years). Acyclovir injection and mecobalamin tablets were used to treat HZ. The patients were also divided into two groups according to the onset of PHN 4 weeks after zoster resolution. The diagnosis of PHN was performed by routine questioning. The diagnostic criteria of PHN were as follows: discretely localized and unilateral (i.e., dermatomal), intermittent, chronic and of sufficient intensity to affect sleep quality and other normal daily activities^12,13^. Twenty-eight age- and gender-matched individuals who received physical examinations served as the controls. The inclusion criteria for the controls were as follows: infection by VZV during childhood and absence of viral reactivation and subsequent presentation of HZ until recruitment into our study. The demographics of the participants are shown in Table 1. The study protocols were approved by the Ethics Committee of Quzhou Hospital of Dermatovenereology (Approval No.: QPY 2009–012). Informed consent was obtained from each subject. All methods were performed in accordance with the relevant guidelines and regulations.

**Table 1:** Demographics of participants.

Variables of interest	Herpes-zoster group(n = 192)	Control group(n = 28)	Old Herpes-zoster group(n = 27)	Control group(n = 14)
Age (years), (mean ± S.D.)	57.69 ± 14.39	55.75 ± 9.78	67.74 ± 7.43	57.29 ± 5.08
Sex (% female)	48.96	53.57	44.44	42.86
CRP(mg/l)	11.43 ± 0.96^▲▲^	0.45 ± 0.05	14.91 ± 9.63^▲▲^	0.46 ± 0. 29
WBC(10^9^/l)	5.66 ± 0.16	5.49 ± 0.31	6.28 ± 2.38	6.46 ± 2.22
Neutrophil (%)	60.81 ± 0.86	58.76 ± 1.02	62.88 ± 13.52	59.83 ± 15.68
Neutrophil(10^9^/l)	3.59 ± 0.13^▲▲^	3.01 ± 0.16	4.34 ± 2.27	4.10 ± 2.19
Lymphocyte (%)	26.46 ± 0.78^▲▲^	30.61 ± 1.05	24.90 ± 10.03	27.15 ± 11.79
Lymphocyte(10^9^/l)	1.54 ± 0.08	1.56 ± 0.13	1.45 ± 0.50	1.59 ± 0.50
Monocyte (%)	10.47 ± 0.32^▲▲^	8.09 ± 0.28	10.47 ± 4.96	11.46 ± 5.79
Monocyte(10^9^/l)	0.59 ± 0.02	0.70 ± 0.11	0.62 ± 0.26	0.72 ± 0.26
History of Herpes-zoster (whether they had been diagnosed at a medical institution)	no	no	no	no
History of Cytomegalovirus (whether they had been diagnosed at a medical institution)	no	no	no	no
History of Epstein Barr virus (whether they had been diagnosed at a medical institution)	no	no	no	no
History of Herpes simplex virus (whether they had been diagnosed at a medical institution)	no	no	no	no
Seroprevalence of Cytomegalovirus (%total group)	55.21	53.57	56.37	54.37
Seroprevalence of Epstein Barr virus (%total group)	45.83	64.29	59.52	55.63
Seroprevalence of Herpes Simplex virus 1 (%total group)	39.06	35.71	44.44	42.86
Seroprevalence of Herpes Simplex virus 2 (%total group)	11.46	10.71	14.81	14.29
Disease duration (days)	3.02 ± 1.26	NA	2.86 ± 1.17	NA
Postherpetic neuralgia (% at 4 weeks)	46.35	NA	50.00	NA

NA: not applicable.

^▲▲^compare with the Control group P < 0.01.

## Methods

Blood samples (5 ml, anticoagulated with EDTA) were collected from each subject in a fasting state between 08:00 am and 09:00 am two days after admission. The blood plasma (2 ml) was subjected to centrifugation (1,500 g, 10 min, at room temperature) and stored at −80 °C until analysis. Plasma CRP levels were determined using a commercial ELISA kit (MSD, Rockville, MD) according to the manufacturer’s instructions. The remaining blood sample (3 ml) was used to determine the total lymphocyte count and blood cell analysis using ABX Pentra DX120 system (Horiba Medical, France) immediately after sample collection.

**Figure 1:**
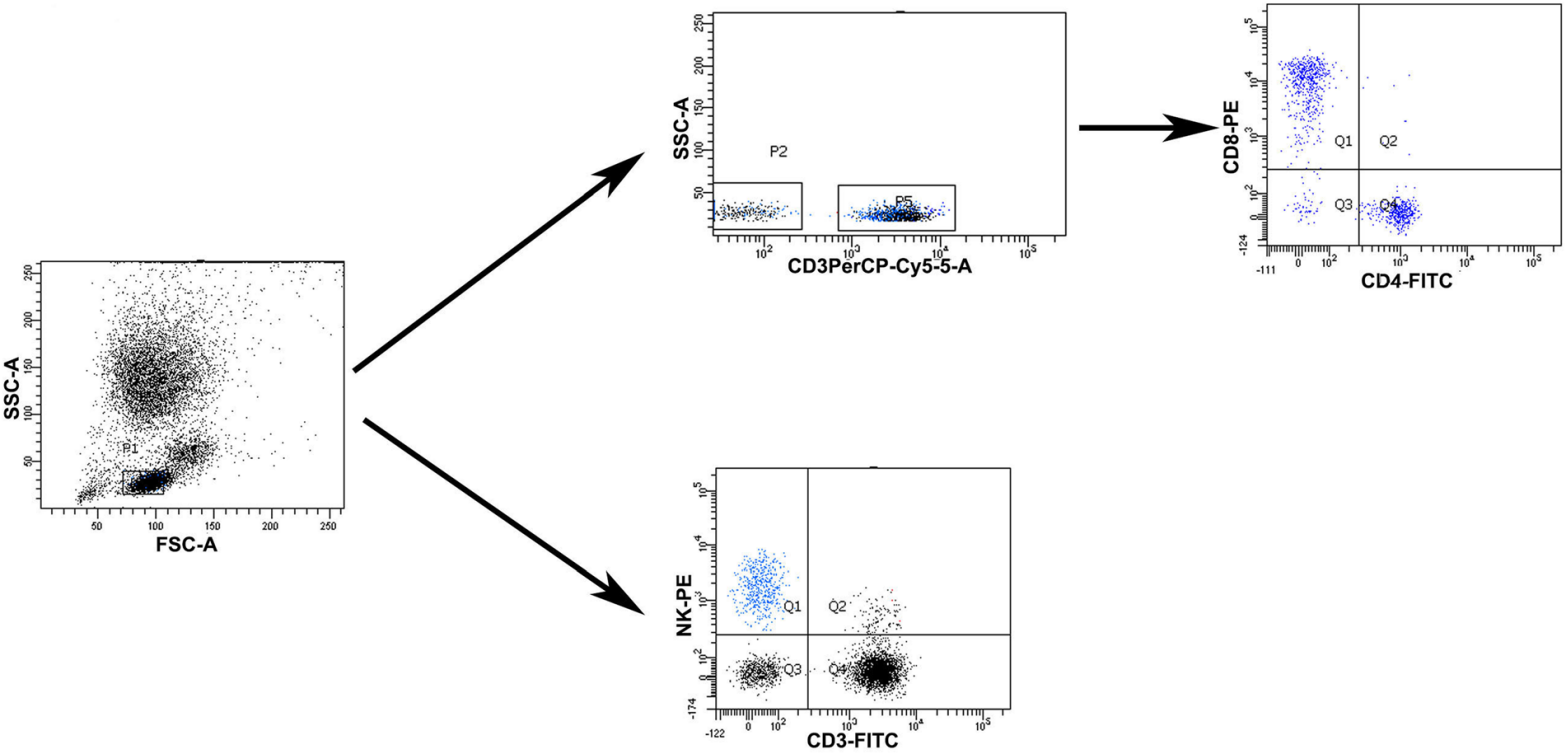
Gating strategy of T lymphocyte subsets and NK cells.

Lymphoid cells were gated for analysis of CD3^+^ T cells, CD4^+^ T cells, CD8^+^ T cells, and natural killer (NK) cells. Initially, whole blood (100 μL) was incubated with antibodies (PerCP-Cy5.5 mouse anti-human CD3, SK7 clone, BD Biosciences; FITC mouse anti-human CD4, SK3 clone, BD Biosciences; PE mouse anti-human CD8, SK1 clone, BD Biosciences; FITC mouse anti-human CD3, UCHT1 clone, Beckman Coulter; PE mouse anti-human CD16, 3G8 clone, Beckman Coulter; PE mouse anti-human CD56, N901 (NKH-1), Beckman Coulter) for 30 min at 4 °C in a dark room. After incubating for 30 min, red blood cells were lysed and the leukocytes were washed in phosphate buffer solution (PBS). Then, all data were acquired immediately on a Beckman Coulter FC500 (Beckman, USA), followed by analysis using MXP software. Gating strategy of different immune cell populations was shown in Fig. 1. Isotype-matched immunoglobulins were used as controls.

**Figure 2:**
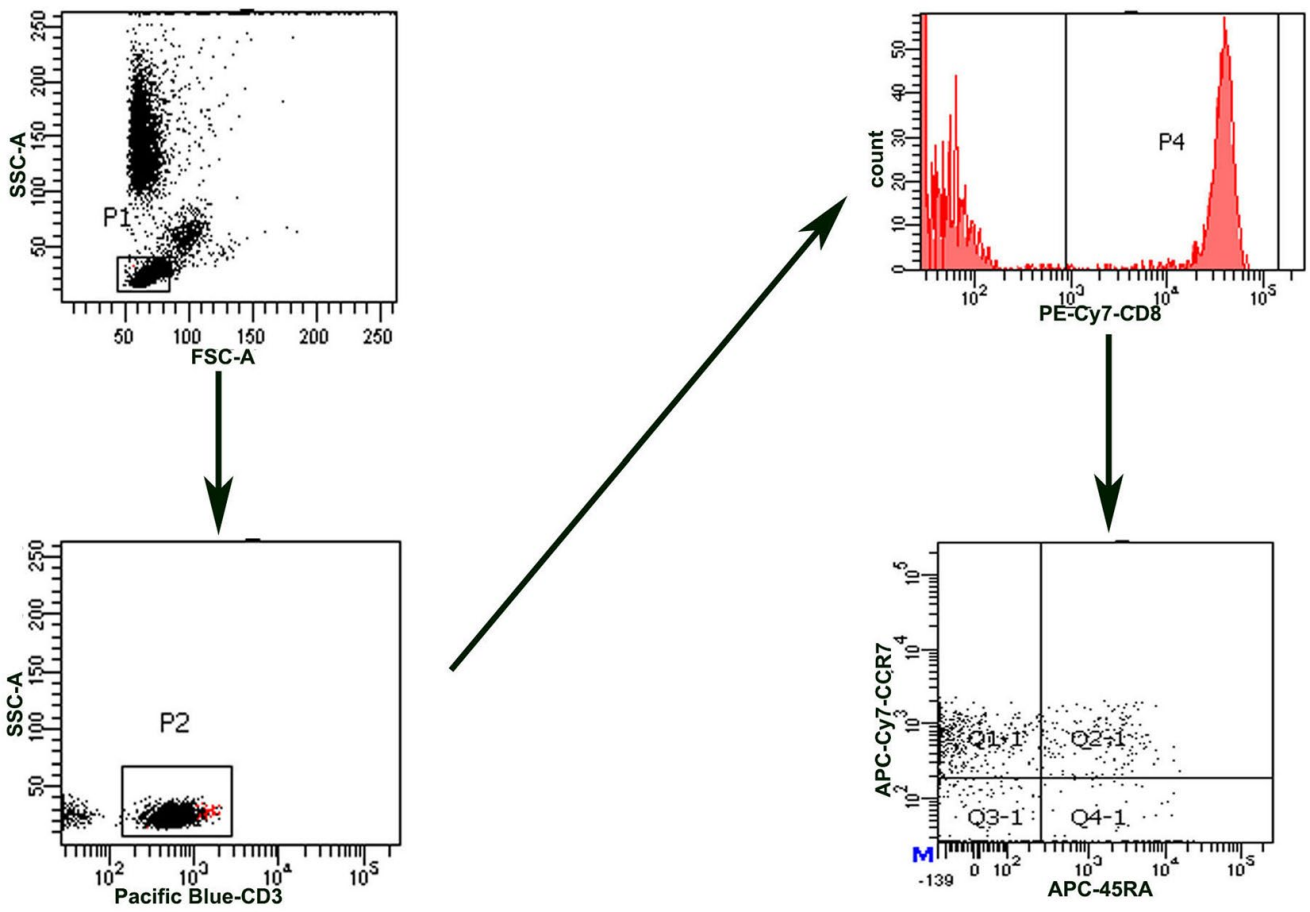
Gating strategy of different phenotypes of CD8 T cells.

Another 5 ml of fasting EDTA-treated venous blood was obtained from the other 24 patients and 14 healthy controls on the second day in the hospital. Lymphoid cells were gated for analysis of CD3, CD8, and other phenotype distributions of CD8^+^ T cells, i.e., T naïve (CD45RA^+^ CCR7^+^), T central memory (TCM; CD45RA^−^ CCR7^+^), T effector memory (TEM; CD45RA^−^ CCR7^−^) and TEMRA (TEMRA; CD45RA^+^ CCR7^−^) populations of CD8^+^ T cells. The antibodies used included Pacific Blue–conjugated anti-human CD3 (UCHT1, BD Bioscience), PE-cy7 conjugated anti-human CD8 (SK1, BD), APC- conjugated anti-human CD45RA (HI100, BD), APC-eflour-780- conjugated anti-human CCR7 (3D12, eBioscience). A total of 100 µl of whole blood was incubated for 30 min at 4 °C in a dark room with monoclonal antibodies. Red blood cells were lysed and the leukocytes were washed in PBS. All data were acquired with a BD FACS Canto II flow cytometer (BD, USA) and analyzed using DIVA software. Gating strategy of different immune cell populations was shown in Fig. 2. Isotype-matched immunoglobulins were used as controls.

For the determination of immunoglobulin and complement proteins, fasting blood samples (5 ml) were collected from each subject in sterile plastic vacutainer tubes without anticoagulant between 08:00 am and 09:00 am two days after admission. The serum was centrifuged for 10 min at 1800 g/min after sample clotting. Immunoglobulins (e.g., IgA, IgG, and IgM) and complement proteins (e.g., C3 and C4) were detected using immunoturbidimetry on an automatic biochemical analyzer (Hitachi 7600, Japan). EBV serology was determined using a commercial ELISA kit (Shenzhen YHLO Biotech Co., Ltd, China) according to the manufacturer’s instructions. CMV serology was determined using a commercial ELISA kit (Medson lnc, USA) according to the manufacturer’s instructions. HSV-1 serology was determined using a commercial ELISA kit (Shanghai fusheng Industrial Co., Ltd, China) according to the manufacturer’s instructions. HSV-2 serology was determined using a commercial ELISA kit (Shanghai keshun Biological Technology Co., Ltd, China) according to the manufacturer’s instructions. The EBV serology, CMV serology, HSV-1 serology, and HSV-2 serology were analyzed with a multimode reader (Bio-RAD iMARK, USA).

### Statistical analysis

Data were analyzed with SPSS 13.0 software and GraphPad Prism 6. All data are presented as the mean ± standard deviation. Descriptive statistics were used to determine whether all data were normally distributed. Student’s t-test was used for the intergroup comparisons. Comparisons between groups were determined with a one-way analysis of variance (ANOVA). The associations between age, immune markers and PHN were examined with Pearson’s correlation coefficients. P < 0.05 was considered to be statistically significant.

## Results

### Demographics of participants

Compared with the controls, we found that the level of CRP and the frequencies of monocyte and neutrophil were significantly lower in HZ patients (P < 0.01). Otherwise, the frequency of lymphocyte was significantly lower in HZ patients than the controls (P < 0.01). However there was no significant difference in the seroprevalences of CMV, EBV, HSV-1, and HSV-2 and the disease duration between HZ group and the control group (P > 0.05, Table 1).

### Absolute numbers of T lymphocyte subsets and NK cells in the HZ group and control group

Compared with the control group, the absolute numbers of CD3^+^ T cells, CD4^+^ T cells and CD8^+^ T cells in the HZ group were significantly lower (P < 0.01). However, there was no significant difference in the absolute number of NK cells between the HZ group and control group (P > 0.05, Table 2).

**Table 2:** Absolute numbers of T lymphocyte subsets and NK cells in the herpes zoster group and control group.

Groups	CD3^+^ T cells (10^9^/l)	CD4^+^ T cells (10^9^/l)	CD8^+^ T cells (10^9^/l)	NK cells (10^9^/l)
Group 1 (n = 192)	0.69 ± 0.04	0.33 ± 0.02	0.31 ± 0.02	0.23 ± 0.01
Group 2 (n = 28)	1.17 ± 0.10	0.59 ± 0.06	0.51 ± 0.04	0.23 ± 0.03
t value	−4.06	−4.49	−3.28	0.06
p value	0.00	0.00	0.00	0.96

Group 1: Herpes zoster group; Group 2: Control group.

### Absolute number of T lymphocyte subsets and NK cells in different age groups of HZ patients

**Figure 3:**
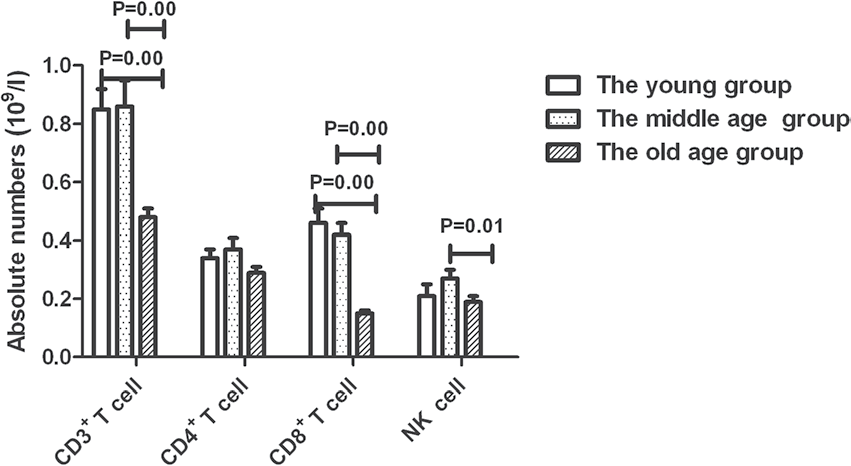
Absolute numbers of T lymphocyte subsets and NK cells in different age groups with herpes zoster.

Compared with the young age group, the absolute numbers of CD3^+^ T cells and CD8^+^ T cells in the old age group were significantly lower (P < 0.01). Compared with the middle age group, the absolute numbers of CD3^+^ T cells, CD8^+^ T cells and NK cells in the old age group were significantly lower (P < 0.05, Fig. 3).

### Serum immunoglobulin and complement proteins in the HZ group and control group

Serum immunoglobulin and complement proteins were similar in the HZ group and the control group, and these are summarized in Table 3.

**Table 3:** Serum immunoglobulin and complement proteins in the herpes zoster group and control group.

Groups	IgG (mg/dl)	IgA (mg/dl)	IgM (mg/dl)	C3 (mg/dl)	C4 (mg/dl)
Group 1 (n = 192)	1225.48 ± 399.24	232.76 ± 114.58	120.14 ± 72.01	78.68 ± 44.98	54.22 ± 44.01
Group 2 (n = 28)	1206.50 ± 288.44	241.77 ± 117.51	105.53 ± 57.55	80.81 ± 40.82	46.82 ± 36.11
t value	0.31	−0.38	1.21	−0.25	0.98
p value	0.76	0.71	0.23	0.80	0.33

Group 1: Herpes zoster group; Group 2: Control group.

### Serum immunoglobulin and complement proteins and plasma CRP in different age groups of HZ patients

Table 4 shows the serum immunoglobulin and complement proteins and plasma CRP levels in different age groups of HZ patients. There were no significant differences in serum immunoglobulin and complement proteins and plasma CRP levels between different age groups of HZ patients.

**Table 4:** Serum immunoglobulin, complement proteins and plasma CRP levels in different age groups with herpes zoster.

Groups	IgG (mg/dl)	IgA (mg/dl)	IgM (mg/dl)	C3 (mg/dl)	C4 (mg/dl)	CRP (mg/l)
Group 1 (n = 25)	1076.32 ± 449.20	203.56 ± 111.81	115.62 ± 68.85	67.36 ± 54.56	63.92 ± 44.24	9.03 ± 2.00
Group 2 (n = 82)	1234.04 ± 429.42	232.39 ± 122.89	122.33 ± 73.42	83.34 ± 44.05	53.93 ± 44.71	10.93 ± 1.47
Group 3 (n = 85)	1260.99 ± 344.52	241.72 ± 106.58	119.34 ± 72.28	77.45 ± 42.64	51.65 ± 43.37	12.63 ± 1.53

Group 1: The young group;

Group 2: The middle age group;

Group 3: The old age group.

### Serum immunoglobulin, complement proteins and plasma CRP in the PHN group and non-PHN group

There were no significant differences in serum immunoglobulin and complement proteins between PHN patients and non-PHN patients. Meanwhile, no significant difference was observed in the plasma CRP levels between PHN patients and non-PHN patients (Table 5).

**Table 5:** Serum immunoglobulin, complement proteins and plasma CRP in the postherpetic and non-postherpetic neuralgia group.

Groups	IgG (mg/dl)	IgA (mg/dl)	IgM (mg/dl)	C3 (mg/dl)	C4 (mg/dl)	CRP (mg/l)
Group 1 (n = 103)	1224.02 ± 434.91	232.59 ± 122.47	126.71 ± 77.57	73.00 ± 44.84	57.43 ± 44.31	11.45 ± 1.21
Group 2 (n = 89)	1227.18 ± 355.31	232.97 ± 105.30	112.47 ± 64.50	85.32 ± 44.47	50.47 ± 43.60	10.59 ± 1.17
t value	−0.06	−0.02	1.37	−1.91	1.10	0.51
p value	0.96	0.98	0.17	0.06	0.28	0.61

Group 1: non-postherpetic neuralgia group;

Group 2: postherpetic neuralgia group.

### Absolute numbers of T lymphocyte subsets and NK cells of PHN and non-PHN patients in different age groups

**Figure 4:**
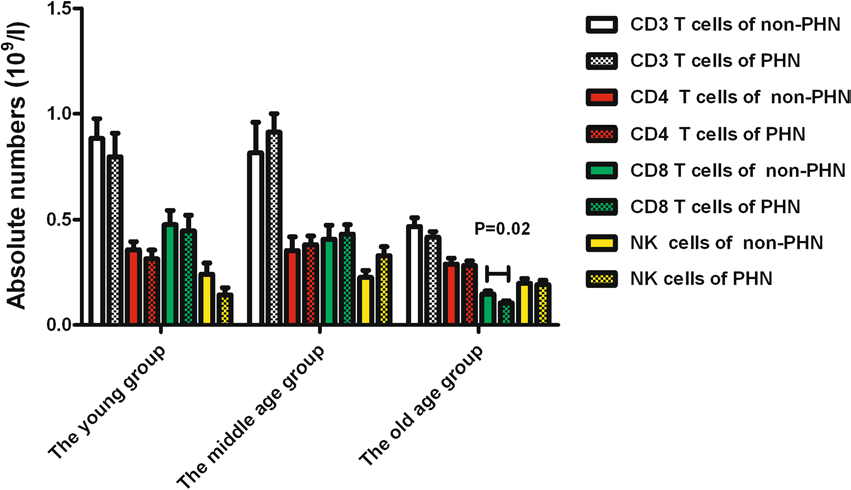
Absolute numbers of T lymphocyte subsets and NK cells of PHN patients and non-PHN patients in three age groups.

Compared with the non-PHN group, the absolute number of CD8^+^ T cells was significantly lower in PHN patients within the old age group (P < 0.05, Fig. 4); however, no significant difference was observed between PHN and non-PHN patients in the young age group and middle age group (Fig. 4).

### Multivariate analysis correlations between age and immune markers with PHN

By multivariate analysis, immune markers, age and CD8^+^ T cells showed significant correlations with PHN (Pearson correlation [age] = 0.20, P = 0.01, Pearson correlation [CD8^+^ T cells] = −0.15, P = 0.04 Table 6).

**Table 6:** Multivariate analyses the correlations of age and immune markers with PHN.

		PHN	Age	CD3	CD4	CD8	NK	CRP	Neutrophil	Lymphocyte	Monocyte
**PHN**	**Pearson**										
	**Correlation**	1.00	0.21	−0.02	0.01	−0.15	0.09	−0.05	−0.10	−0.04	0.07
	**P value**		0.00	0.81	0.87	0.04	0.23	0.49	0.16	0.55	0.35
**Age**	**Pearson**										
	**Correlation**	0.21	1.00	−0.32	−0.16	−0.45	−0.08	0.07	−0.14	0.00	−0.07
	**P value**	0.00		0.00	0.02	0.00	0.25	0.34	0.05	0.97	0.35

### Quantitative analysis of different phenotypes of CD8^+^ T cells of old age PHN and non-PHN patients (HZ patients and controls)

**Figure 5:**
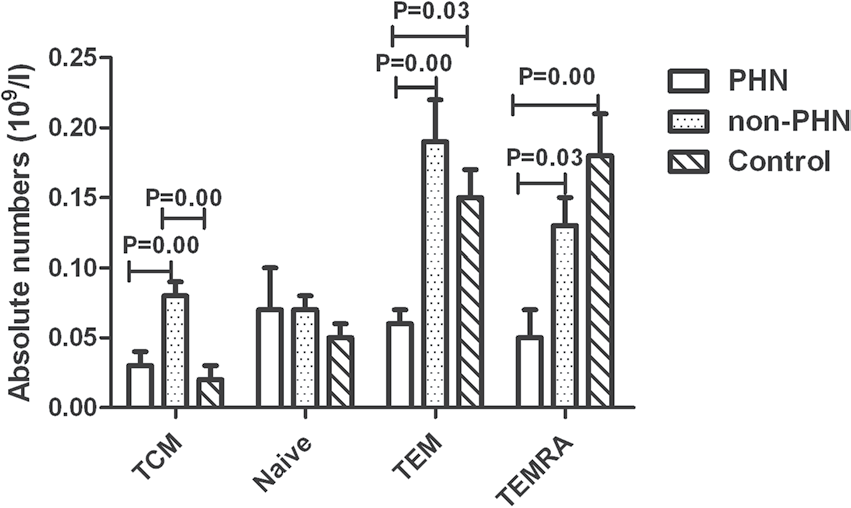
Absolute numbers of different phenotypes of CD8 T cells in PHN patients and non-PHN patients for old age HZ patients and controls.

Compared with the control group, the absolute numbers of CD8^+^ TEM cells and CD8^+^ TEMRA cells were significantly lower in the PHN group (P < 0.05); however, the absolute numbers of CD8^+^ TCM cells was significantly higher in the non-PHN group (P < 0.01). Compared with the non-PHN group, the absolute numbers of CD8^+^ TCM, CD8^+^ TEM and CD8^+^ TEMRA cells were significantly lower in the PHN group (P < 0.05, Fig. 5).

## Discussion

We systematically investigated the characteristics of immune responses during aging and PHN in HZ patients by analyzing T lymphocyte subsets, immunoglobulins and complement proteins. Our data show that immune responses decreased during aging and PHN pathogenesis.

Some study reported that immune responses and the response speed of VZV affected the spread of the virus^14^. Numerous studies have indicated that immune responses play an important role as the major cause of the disease. To date, the roles of humoral and cellular immunity in VZV reactivation are controversial^15–18^. Rare reports have focused on the features of humoral immunity and cell immunity of HZ patients. Therefore, we selected immunoglobulin and complement proteins as the markers for humoral immunity, and at the same time, T lymphocyte subsets and NK cells were selected as the markers for cell immunity, to systemically study the characteristics of immune responses in the peripheral blood of HZ patients.

Compared with the immunological state in controls, we found that the level of CRP and the frequencies of monocyte and neutrophil were significantly higher in HZ patients. Otherwise, the frequency of lymphocyte was significantly lower in HZ patients than the controls. These indicated that the immune status of the patients changed after the reactivation of VZV. However there was no significant difference in the seroprevalences of CMV, EBV, HSV-1, and HSV-2 and the disease duration between HZ group and the control group.

In this study, there was no significant difference in serum immunoglobulin and complement proteins between the HZ group and control group. However, the absolute numbers of CD3^+^ T cells, CD4^+^ T cells and CD8^+^ T cells in HZ patients were significantly lower compared to the controls. These results indicate that cell-mediated immunity may be important during the reactivation of VZV, probably due to the spread of the virus within the body exclusively via the intracellular route^19–22^.

T lymphocyte subsets in the peripheral blood are considered to be the main parameters of cell-mediated immunity^23^. T lymphocyte subset interactions are assumed to be in balance to ensure normal immune function. In the case of aberrant changes in the number of lymphocytes, the immune system becomes disordered, and there is a series of pathological changes, which ultimately increases the potential for infection in the body^24^.

The absolute number of CD3^+^ T cells in the peripheral blood of HZ patients was lower, especially in older age patients. The results demonstrated that the T cell were suppressed in old age patients after VZV reactivation, which coincided with the fact that T cell immune suppression was associated with worsened recurrent infections^21^.

Meanwhile, we found that the absolute number of CD8^+^ T cells was obvious lower in the old age group compared to the young group and the middle age group. However, there was no significant difference between the absolute number of CD4^+^ T cells in different age groups of HZ patients, which indicated that the CD8^+^ T cells played important roles after the reactivation of HZ in old age patients^25,26^. The role of CD8^+^ T cells in the control of VZV replication could also be inferred from the accumulation of the T cell subset in the ganglia of individuals with HZ, where VZV was actively replicating^27,28^. Therefore, we speculated that the lower levels of CD3^+^ T cells and CD8^+^ T cells may be the result of the high reactivation of VZV in the old age population.

NK cells, as a part of the innate immune system, are important in defense against infection and cancer. After activation, NK cells are capable of mediating immune function by directly killing target cells^29^. NK cells have been shown to play important roles in controlling persistent HSV-1 infections^30^. In a case report, Etzioni *et al*. emphasized the role of NK cells in controlling HZ infection^31^. Our data indicate that the absolute number of NK cells was obvious lower in old age HZ patients compared to the middle age group, implying that NK cells play a role after VZV reactivation in old age HZ patients.

PHN is a ubiquitous sequelae after HZ infection that severely affects patient quality of life^32^. To date, the mechanism involved in the development of PHN remains unknown. We found that the absolute number of CD8^+^ T cells was significantly lower in PHN patients compared with the non-PHN group for old age patients; however, no significant difference was observed between PHN and non-PHN in the young age group and middle age group. The results showed that the decreased absolute number of CD8^+^ T cells may contribute to immune pathogenesis in the development of PHN in old age patients. Until now, there has been no index to predict PHN in clinical practice. Therefore, we analyzed the correlations of immune markers with PHN, and we found that age and CD8^+^ T cells showed significant correlations with PHN development.

Additionally, to further explore the mechanism of the lower level of CD8 T cells in old age HZ patients, especially in those who developed PHN, we studied the distribution of naïve, TCM, TEM, and TEMRA populations within CD8^+^ T cells in the PHN and non-PHN groups of old age HZ patients. Our data showed that the absolute numbers of CD8^+^ TEM cells and CD8^+^ TEMRA cells were significantly lower in the PHN group compared to the control group; however, the absolute numbers of CD8 TCM cells was significantly higher in the non-PHN group compared to the control group, indicating that the absolute numbers of CD8^+^ memory cells and CD8^+^ TEMRA cells changed in HZ patients. Memory T cells are antigen experienced and can enter lymph nodes through high endothelial venules. These cells can proliferate vigorously in response to antigen stimulation, and some of these cells can differentiate into effector cells^33,34^. TEMRA cells are known to have a superior capacity to respond quickly to antigenic stimulation compared to naïve T cells^35,36^. However, in humans, the developmental relationship among TCM, TEM, and TEMRA is still controversial. Interestingly, we found that the absolute number of CD8^+^ TCM, CD8^+^ TEM and CD8^+^ TEMRA cells in the PHN group were significantly lower compared to the non-PHN group, indicating that the CD8^+^ TCM, CD8^+^ TEM and CD8^+^ TEMRA cells may play important roles in the development of PHN in old age HZ patients. To our knowledge, this is the first report on the absolute numbers of CD8^+^ TCM, CD8^+^ TEM and CD8^+^ TEMRA cells in old age HZ patients who developed PHN, and this may provide a reference for clinical intervention to prevent the development of PHN in old age HZ patients.

In summary, we found that the absolute numbers of CD3^+^ T cells and CD8^+^ T cells were lower during aging, especially in old age HZ patients who develop PHN. Meanwhile, CD8 T cells play important role in the development of PHN in old HZ patients, which may provide reference for clinic to prevent the development of PHN in old age HZ patients.
